# Claim causality with clarity

**DOI:** 10.1093/psyrad/kkad007

**Published:** 2023-06-09

**Authors:** Qing Wang, Qiao Wang, Ru-Yuan Zhang

**Affiliations:** Shanghai Mental Health Center, School of Medicine, Shanghai Jiao Tong University, 600 S. Wanping Road, Shanghai 200030, Shanghai, China; Department of Biostatistics and Bioinformatics, Duke University, 2424 Erwin Road, Durham NC, 27705, USA; Shanghai Mental Health Center, School of Medicine, Shanghai Jiao Tong University, 600 S. Wanping Road, Shanghai 200030, Shanghai, China; Institute of Psychology and Behavioral Science, Antai College of Economics and Management, Shanghai Jiao Tong University, Shanghai 200030, China

*We do not have knowledge of a thing until we have grasped its why, that is to say, its cause*.

*— Aristotle, Physics*

Causality and identification of the underlying mechanisms are the goals of scientific research. Many researchers devote their efforts to the formal formulation of causal inference to bridge the philosophical thinking and theoretical foundation for applied sciences, e.g. medical and health research, so that causal conclusions can be made. Nevertheless, the lure of fancy causal claims without clarity and rigorous sensitivity analysis can be misleading (Mehler & Kording, 2020). In this short review, we introduce the statistical basics for causal inference and the most commonly used methods to estimate causal effects, and provide suggestions for conducting open and reproducible causal analyses with clarity.

## The Rising Trend of Causal Related Research

**Figure 1:**
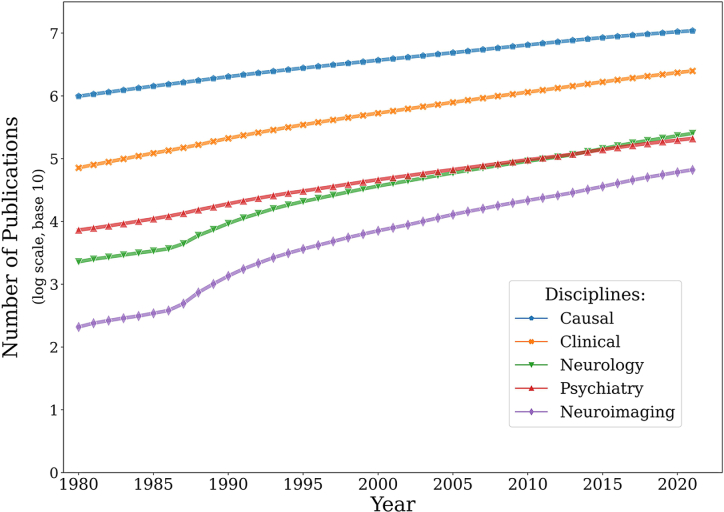
Causal related research literature from PubMed search.

Causal inference is one of the most popular topics in statistics, and its applications in both experimental and observational research have exponentially grown. Figure 1 shows the number of publications related to causal research as an indicator of its popularity in different research disciplines. The data are obtained from a PubMed search using expressions such as “(causal OR causality) AND (discipline)” based on the texts of publication without further manual content validation, the full details of which are available on the GitHub repo: https://github.com/Vincent-wq/causal_literature_trend. As illustrated in Fig. 1, causal related research has the richest literature and the largest number of published papers. Clinical related causal research has the second largest number of papers published. Both neurology and psychiatry show similar escalating trends. Interestingly, the rising slope of neurology exceeded that of psychiatry in 2014, which may indicate that the application of causal related analysis has become more widespread in neurology than in psychiatry. However, it is hard to know the reasons for such changes without a detailed in-depth literature review. Neuroimaging has recently enjoyed a burst of applications in clinical practice, especially in neurology and psychiatry, yet it has the smallest number of published papers. This may be related to the complexity and high-dimensional nature of neuroimaging data and modeling. In conclusion, the number of causal related publications is increasing.

The misuse and misinterpretation of statistical methods have contributed to the reproducibility crisis (Adler *et al*., n.d.; Baker, 2016; Open Science Collaboration, 2015; Wang *et al*., 2023). By analogy, the boosting of causal related research calls for better communication and interpretation of causal analysis. The aim of this mini review is to raise the awareness of the clarity when reporting and interpreting causal related research so that the misuse and misinterpretation can be reduced.

## Basics of Causal Inference

In this review, we limit our discussion of causal inference to the evaluation of causal effects rather than the identification of causal mechanisms. Causal inference can be conducted by (i) formulating the research question in a causal framework; (ii) specifying assumptions based on which causal effects can be identified; and (iii) assessing the sensitivity to the violation of causal assumptions. There are two main causal inference frameworks: the potential outcome (PO) framework (Hernán & Robins, 2020) and the causal diagram framework (Judea Pearl, 2009). These two frameworks are mathematically connected with different established goals (Richardson & Robins, 2013). We will focus on the PO framework in this review as most of the literature reviewed falls under the umbrella of the PO framework.

**Figure 2:**
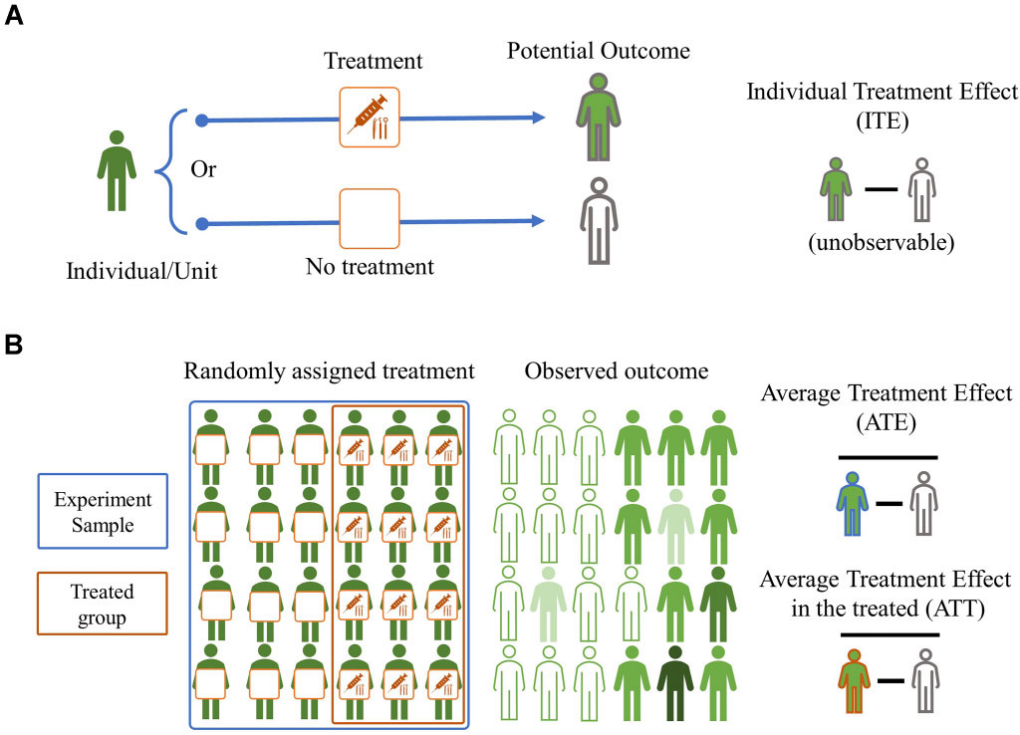
Visual illustrations of the basic concepts in causal inference. (A) ITE. Since the individual cannot simultaneously receive and not receive the treatment, we are unable to observe the difference between the POs (icon with boundary) of receiving treatment and not receiving treatment for the same individual, i.e. ITE is unobservable; (B) ATE and ATT. ATE is the average treatment effect for the whole group while ATT is the average treatment effect for the treated group, ATE = ATT for the ideal RCT (being in the control or treatment group is random and unrelated to the outcome), but they are not necessarily the same in the observational studies. We use the observed outcome to estimate ATE and ATT.

First, we briefly review the key concepts in the PO framework as illustrated in Fig. 2: (i) unit, the person or subject on whom the treatment will be operated; (ii) target population, a well-defined population of units whose causal effects are going to be estimated; (iii) sample, a random sample of *N* from the target population, the data collected from the sample being used for further analysis; (iv) treatment (intervention/exposure/manipulation), the effects of which the investigator would like to assess compared to no such treatment; and (v) outcome, the final observation after treatment (can be no treatment). The PO framework aims to answer the question “what would potentially happen to the same units or participants had they exposed to a different (counterfactual) condition (treatment)?” By definition, we can never observe the individual treatment effect (ITE) since we can only observe the outcome from one treatment at a time (illustrated in Fig. 2A). Most of the time, the average treatment effect (ATE) or average treatment effect in the treated (ATT) is the main causal effect we would like to estimate (as illustrated in Fig. 2B). Stated formally, causal inference is to estimate the causal effect from the outcome of a treatment, intervention, exposure, or manipulation with observed confounders and/or covariates and unobserved confounders and/or covariates.

Traditional statistical inference draws conclusions based on associations, and the main differences between these traditional data analyses and causal inference lies in the causal assumptions, i.e. the identification conditions for causal effects. One basic assumption for causal inference is the stable unit treatment value assumption (SUTVA): “The potential outcomes for any unit do not vary with the treatments assigned to other units, and, for each unit, there are no different forms or versions of each treatment level, which lead to different potential outcomes” (Imbens & Rubin, 2015). SUTVA describes the basic properties of treatment unite and connects the intervention we observed with the causal intervention of interest, and it is a strong assumption about no interference and no multiple versions of a treatment, which contributes to a well-defined intervention.

## Causality in Experimental and Observational Research

The golden standard of estimating causal effects is the ideal randomized controlled trial (RCT) (Hernán & Robins, 2020), where RCT is a true random sample from the target population. In addition to the SUTVA, an ideal RCT with well-established random treatment assignment mechanisms allows the inference of causal effects since it satisfies the following assumptions: (i) “Unconfoundedness” (or “Ignorability,” “Exchangeability”), (ii) “Positivity” (or “overlap”), and (iii) “Consistency” (part of the SUTVA assumption) (Cole & Frangakis, 2009). Specifically, Unconfoundedness assumes the independence of treatment assignment and the outcomes, which implies that within the subpopulations defined by the values of observed covariates, the treatment assignment is random, i.e. treated and untreated participants, censored and uncensored participants have equal distributions of POs. Consistency assumes that an individual's PO under the observed exposure history is precisely the observed outcome. Positivity assumes that all the levels of exposure for every combination of values of exposure and confounders occur among individuals in the population. However, these assumptions cannot always be met, and the ideal RCT can be compromised due to ethical, economical, protocol violations, and other limitations that endanger the estimation of causal effect. Therefore, clarifying causal assumptions and constructing a meaningful causal estimand to draw interpretable causal conclusions is highly challenging, especially for observational studies (Liu *et al*., 2021).

In observational studies, we can neither control nor be clear about the intervention assignment mechanisms, and it is common to violate some or all of the assumptions from before, which makes justification of causal assumptions essential. For example, assuming there are no unobserved confounders, failure in randomized assignment of the treatment may cause imbalanced covariates between the treatment and control groups. As a result, statistical methods must be introduced to balance these two groups, and the typical procedures include regression, matching, propensity score-based methods (such as inverse probability weighting) or their combination such as double robust (DR) estimators (Li *et al*., 2018). When researchers are not confident that all confounders are fully observed and correctly measured, instrumental variable techniques are introduced to circumvent these limitations. (Marinescu *et al*., 2018; Liu *et al*., 2021).

The key logic of causal inference in observational studies is to mimic a target experiment (trial) that produces similar results to an RCT in a hypothesized population. For example, quasi-experimental approaches have been widely used in economics and psychology. Liu, Marinescu and others have reviewed this family of methods including regression discontinuity design, difference in difference, and instrumental variables (IV) (Liu *et al*., 2021; Marinescu *et al*., 2018). An IV is a variable that is only associated with the exposure to the intervention but not with other factors associated with the outcome of interest. Using IV does not require the assumption of unconfoundedness, but three other conditions should be met: namely, the relevance condition, the exclusion restriction, and the marginal exchangeability (Hernán & Robins, 2020). Regression discontinuity design is a special case of IV that uses the discontinuity feature of the running variable as IV. Another commonly used IV in life sciences is genetics, which is assumed to be randomly inherited from the parents, and the corresponding approach is called Mendelian randomization (Burgess & Thompson, 2021). All these models and approaches rely heavily on strong assumptions and complex computations, which means the results can be very different on any meaningful violation of assumptions or any changes in the algorithms or computing environment. Sensitivity analysis is also necessary to assess such biases.

## Causality in Clinical Neuroscience

The current causal inference framework from the statistics world has not been properly translated to face the challenges in clinical neuroscience research due to its intrinsic complexity including but not limited to the lack of RCT data sources due to ethical concerns or other factors such as cost, the justifications of causal assumptions for experiments other than an ideal RCT or observational studies, and the definition of an intervention, which is more complicated than just taking or not taking a specific medicine, and it can be one of many types of brain stimulation, modulation, or even targeted surgery. In addition, Barack *et al*. have called for more clarity about causality in neuroscience research since the word “causality” can refer to as different meanings in neuroscience (Barack *et al*., 2022), some neuroscientists believed that causes are the events that produce other events while others may think that causes are the factors that events depend on. Such ambiguous definitions of “causes” impedes the communication and interpretation of causal analyses from different researchers. Taking clinical research as an example, Siddiqi *et al*. have reviewed most of the available interventions in clinical neuroscience practice regarding mapping human brain functions and have brought about six criteria for appraising causality adapted from Bradford Hill criteria: counterfactual, specificity, experimental manipulation, dose–response relationship, coherence, and reversibility (Siddiqi *et al*., 2022). They also suggested that causal claims based on purely correlation results should be avoided. There are various types of intervention used in clinical neuroscience, such as drugs, non-invasive neuroimaging with stimuli, neurofeedback, lesion, brain stimulation, etc. It is not easy to model all of these interventions with a unified causal framework so that they are comparable, a binary variable (whether to use or not to use a specific type of intervention) is insufficient to capture the full information of these interventions (SUTVA assumption is very likely to be violated); a multivariate mechanistic approach might be helpful, such as dynamic causal modeling (Friston *et al*., 2003), which tries to capture the complex mechanism of how the specific intervention (experimental task design) changes the outcome with a dynamical biophysical forward model. Reid *et al*. attempted to formulate functional connectivity estimates using a causal framework but ended up by using vague definitions and mixing different levels of concepts (Reid *et al*., 2019). For example, there is no clear definition of the “causal effect of interest,” but a rather general term “target theoretical properties” was used. The definition of “confounding properties” mainly includes artifacts during the imperfect measurement of functional connectivity, but there are so many more confounding sources outside the measurement procedure, such as age, sex, and so on. Another emerging trend is the mining of large-scale observational imaging datasets and imaging-derived phenotypes with the Mendelian randomization approach, i.e. using genome as an instrumental variable to evaluate the potential causal relationships between imaging-derived phenotypes and neurological or psychiatric disorders (Guo *et al*., 2022; Taschler *et al*., 2022). In summary, solid statistical-based causal inference is still lacking in clinical neuroscience research, and we are still at the stage of formulating the questions properly with causal language, where the process can be benefited by interdisciplinary collaborations.

## Claim Causality with Clarity

Clarity and sensitivity analyses are crucial for causal inference, especially in observational studies. To promote open and reproducible research (Jin *et al*., 2022) and to avoid further mis-claiming or misinterpretation of causal analysis, we encourage the researchers to report: (i) full details of the causal formulation of the research question and the reasoning behind the causal model (can be represented by a directed acyclic graph or DAG), including: (a) study type (whether this is a RCT or observational study), (b) well-defined causal effect of interest (e.g. ATE or ATT), including clear descriptions of treatment/intervention/exposure/manipulation, confounder selection and its rationale, known unobserved confounders with corresponding assumptions about them, and (c) the observed outcome (continuous or binary, etc.); (ii) all the necessary assumptions condition on which the causation can be interpretated, especially for observational studies, e.g. whether Unconfoundedness, positivity, and consistency are reasonable assumptions for this study; (iii) full details of the causal estimand, including (a) the statistical approach and (b) the effect size of the causal effect, such as the estimation of ATE or ATT or causal odds ratios; (iv) the results of sensitivity analysis, for both the meaningful violations of the assumptions in (ii) and different model estimation algorithms. With all necessary information shared, readers and reviewers should be able to replicate and generalize such causal analyses and have a better understanding of the strength of the causal claims.

## Summary

In this mini review, we started with a simple literature search on causal analyses and showed its exponential accumulation and similar increasing trends in clinical research, neurology, and psychiatry. We introduced the basic ideas and the concepts of causal inference under the PO framework and explained the key differences of causal inference in RCT and observational studies. We also reviewed the most recent literature on causal analysis in clinical neuroscience and the related neuroimaging studies, it is fruitful, yet more efforts are still needed for formal causal formulation and interpretation. We conclude this review with four recommendations for conducting open and reproducible causal inference research.

## Supplementary Material

kkad007_Supplemental_File
