# Supplementary material for: Claim causality with clarity
Source: Psychoradiology. 2023 Jun 9;3:kkad007. doi: 10.1093/psyrad/kkad007 (PMC10917363; doi:10.1093/psyrad/kkad007)
Supplement: kkad007_Supplemental_File [file kkad007_Supplemental_File.docx]

Dear editor,

Thank you for the following up comments on our manuscript entitled *Claim* *Causality with Clarity*. The main concerns of the reviewers are:

1) The title of section 1 should better match the revised content.
1) Some typos should be fixed.

Detailed inline replies are provided below for the reviewers. All changes are marked in red in the revised manuscript. We look forward to further communications.

B. R.

Qing Wang, Qiao Wang and Ru-Yuan Zhang

Reviewer: 3
Comments to the Author

The authors followed my recommendations and suggestions and the manuscript has been improved in many respects. The added Figure 1 makes the concepts described in the manuscript better accessible.

1) However, I still have concerns about Chapter 1 of their manuscript. As it stands now, there is a mismatch between the title 'Causality is the future' and the author's aim for this chapter as they write "The only point we would like to make from the literature search is that the increasing of causal related research requires better communication of causal analysis"

Moreover, causality is not the goal of all studies in my opinion. It would be beneficial if the authors can find a title that matches the content of the section.
Response: We have updated the title for this section as “the rising trend of causal related research” to better match the revised content.

We further add details for the PubMed search description after a brief communication with the editors: “The data are obtained from a PubMed search using expressions such as “(causal OR causality) AND (discipline)” based on the publication texts without further manual content validation,” in Line 43.

2) Typo: line 173 dose-response relationship
Response: The typo has been fixed and the entire manuscript has been checked for other typos as well.
